# *In Silico* Analysis of Bioethanol Overproduction by Genetically Modified Microorganisms in Coculture Fermentation

**DOI:** 10.1155/2015/238082

**Published:** 2015-02-16

**Authors:** Lisha K. Parambil, Debasis Sarkar

**Affiliations:** Department of Chemical Engineering, Indian Institute of Technology Kharagpur, Kharagpur 721 302, India

## Abstract

Lignocellulosic biomass is an attractive sustainable carbon source for fermentative production of bioethanol. In this context, use of microbial consortia consisting of substrate-selective microbes is advantageous as it eliminates the negative impacts of glucose catabolite repression. In this study, a detailed *in silico* analysis of bioethanol production from glucose-xylose mixtures of various compositions by coculture fermentation of xylose-selective *Escherichia coli* strain ZSC113 and glucose-selective wild-type *Saccharomyces cerevisiae* is presented. Dynamic flux balance models based on available genome-scale metabolic networks of the microorganisms have been used to analyze bioethanol production and the maximization of ethanol productivity is addressed by computing optimal aerobic-anaerobic switching times. A set of genetic engineering strategies for ethanol overproduction by *E. coli* strain ZSC113 have been evaluated for their efficiency in the context of batch coculture process. Finally, simulations are carried out to determine the pairs of genetically modified *E. coli* strain ZSC113 and *S. cerevisiae* that significantly enhance ethanol productivity in batch coculture fermentation.

## 1. Introduction

Environmental concerns and energy security issues have renewed our interest in bioethanol as a substitute for petroleum derived liquid transportation fuel. Bioethanol is mainly produced from edible starch crops or sugar cane. The use of this first generation feedstock is uneconomical and leads to food versus fuel dispute. Lignocellulosic biomasses, the most abundant biological material on earth, are an attractive alternative feedstock for bioethanol production. It essentially contains cellulose (~45% of dry weight), hemicellulose (~30% of dry weight), and lignin (~25% of dry weight) [1]. Hydrolysis of cellulose produces easily fermentable hexose sugar (glucose) and hydrolysis of hemicellulose produces a mixture of hexose (glucose) and pentose sugars (xylose, arabinose). Due to this complex composition, the commercial utilization of lignocelluloses as bioethanol feedstock faces many technical and economic challenges. The proper selection of microorganisms for the fermentation step is thus very important. The productivity of the fermentation step can be enhanced by genetic manipulation of traditional strains for consumption of both glucose and xylose [2, 3] or by carrying out coculture fermentation of specialized microbes [4, 5]. This second alternative is particularly advantageous as it leads to simultaneous consumption of both glucose and xylose sugars.

Genome-scale metabolic networks are now available for a number of organisms and the availability of these models offers new approaches to improve the understanding of complex biological processes. A successful approach to genome-scale modelling is the constraint-based modelling approach which attempts to explore feasible phenotypes of an organism at given pseudo steady-state condition. Flux balance analysis (FBA) is an efficient constraint-based approach to analyze a genome-scale metabolic network. It uses linear programming to determine the intracellular fluxes that optimize a given objective function [6, 7]. Most of the modelling techniques that have been developed for systemic understanding of cellular functions require detailed information regarding reaction kinetics as well as enzyme and metabolite concentrations. But FBA requires minimal amount of biological knowledge and kinetic data to make quantitative predictions about metabolic phenotype [8]. The dynamic flux balance analysis (dFBA) models are obtained by combining stoichiometric equations for intracellular metabolism with dynamic mass balances on key extracellular substrates/products under the assumption of fast intracellular dynamics and are applicable for accounting the unsteady-state situation in batch/fed-batch fermentation [9].

Microbial coculture (fermentation with two or more microorganisms) appears to be advantageous over single-organism culture for bioethanol production from lignocelluloses due to the potential of synergistic utilization of metabolic capabilities of involved microbes for the cofermentation of glucose and xylose [5]. The superiority of coculture of substrate-selective microbes (engineered* Escherichia coli* and wild-type* Saccharomyces cerevisiae*) over single-organism culture (recombinant* S. cerevisiae* RWB218) in improving the utilization of glucose/xylose mixtures for enhanced bioethanol production from batch fermentation using dFBA modelling technique has been reported by Hanly and Henson [10] and Hanly et al. [11]. By using the genome-scale metabolic network of* S. cerevisiae* (*i*FF708), Bro et al. [12] reported ten genetic engineering strategies for enhancing ethanol yield at the expense of reduced glycerol production. Recently, Lisha and Sarkar [13, 14] analysed the impact of ten genetic engineering strategies reported by Bro et al. [12] on* S. cerevisiae* for their efficiency in enhancing the ethanol productivity in the context of batch/fed-batch coculture and monoculture fermentation. Simulations were carried out with various glucose/xylose mixtures and, for the 50/50 glucose/xylose (%/%) mixture, the batch coculture fermentation using genetically modified* S. cerevisiae* (consumes only glucose) and engineered* E. coli* strain ZSC113 (consumes only xylose) enhanced the ethanol productivity by 40.7% compared to the monoculture (*S. cerevisiae* strain RWB218) fermentation. The enhancement in ethanol productivity of coculture system of substrate-selective microbes is due to the simultaneous conversion of both glucose and xylose sugars, high substrate utilization rate, and reduced fermentation time compared to monoculture system of* S. cerevisiae* strain RWB218. The authors suggested that genetic modification on xylose-selective* E. coli* strain ZSC113 should also be explored as an alternative approach for enhanced bioethanol production from coculture system. Bioethanol production potential of* Scheffersomyces *(*Pichia*)* stipitis* from glucose/xylose mixtures has been investigated using dFBA analysis [15, 16].

The objective of the present study is to conduct* in silico* analysis of the effect of various genetic engineering strategies on xylose-selective* E. coli* strain ZSC113 towards enhanced production of ethanol from glucose/xylose mixtures in batch coculture fermentation with wild-type* S. cerevisiae*. Next, pairs of genetically modified* E. coli* strain ZSC113 and* S. cerevisiae* are determined through simulations of genome-scale models, which significantly enhance ethanol production. Batch ethanol productivity is taken as a measure of fermentation performance and the maximization of ethanol productivity is sought with respect to the optimal aerobic to anaerobic switching time. In order to investigate the effect of various lignocellulosic feedstocks that contain glucose/xylose mixture in varying proportions, simulations are carried out with 50/50, 60/40, and 70/30 glucose/xylose (weight%/weight%) mixtures.

## 2. Methods

### 2.1. Dynamic Flux Balance Model

The stoichiometric models used in the present study are adapted from* i*ND750* S. cerevisiae* [17] and* i*AF1260* E. coli* [18] genome-scale metabolic networks. The wild-type* S. cerevisiae* model* i*ND750 is a fully compartmentalized genome-scale metabolic network with 750 genes and 1150 intracellular reactions. From 1061 metabolites and 1266 fluxes, which include 116 membrane exchange fluxes, the stoichiometric matrix of size 1061 × 1266 is formed. The wild-type* E. coli* model* i*AF1260 consists of 1261 genes and 2083 intracellular reactions. The dimensions of the stoichiometric matrix for model* i*AF1260 are 1668 × 2382 with 299 membrane exchange fluxes. To simulate xylose-selective* E. coli* strain ZSC113, the glucose exchange and glucokinase fluxes are constrained to zero in* i*AF1260. If two microorganisms are assumed to be noninteracting and each species maximizes its own growth rate using available resources, the model for the coculture system can be developed by combining the dFBA models for individual species [10]. The standard linear program to solve the underdetermined flux balance model of coculture system of two microbial species, glucose-selective* S. cerevisiae* (SC) and xylose-selective* E. coli* strain ZSC113 (EC), can thus be formulated as follows:

(1) MaximizevSC,vEC  μ=μSC+μEC=wSCTvSC+wECTvECSubject  to:    ASC00AECvSCvEC=00      vSC,min⁡vEC,min⁡≤vSCvEC≤vSC,max⁡vEC,max⁡,

where *A* is the matrix of stoichiometric coefficients, *v* is vector of reaction fluxes including exchange fluxes, *μ* is the cellular growth rate, and *w* is a vector of weights that represent the contribution of each flux to cellmass formation. The stoichiometric matrix *A* is the mathematical representation of the reaction list. It is an *m* × *n* matrix where *m* is the number of metabolites and *n* is the number of reactions. Each element of *A* (*A*_*ij*_) represents the stoichiometric coefficient of the *i*th metabolite in the *j*th reaction. The coefficient is positive when the metabolite is a product of the given reaction and negative when the metabolite is a substrate.

Substrates uptake kinetics for the microorganisms are modelled as Michaelis-Menten kinetics with additional regulatory term to account for growth rate suppression at high ethanol concentration:

(2) $$\begin{array}{c} v_{\text{g},\text{SC}}=v_{\text{g},\max,\text{SC}}\frac{G}{K_{\text{g},\text{SC}}+G}\frac{1}{1+E/K_{\text{ie},\text{SC}}},\\ v_{\text{z},\text{EC}}=v_{\text{z},\max,\text{EC}}\frac{Z}{K_{\text{z},\text{EC}}+Z}\frac{1}{1+E/K_{\text{ie},\text{EC}}},\\ v_{\text{o},\text{SC}}=v_{\text{o},\max,\text{SC}}\frac{O}{K_{\text{o},\text{SC}}+O},\\ v_{\text{o},\text{EC}}=v_{\text{o},\max,\text{EC}}\frac{O}{K_{\text{o},\text{EC}}+O}, \end{array}$$

where *G*, *Z*, *E*, and *O* are the glucose, xylose, ethanol, and dissolved oxygen concentrations, respectively. *v*_g_, *v*_z_, and *v*_o_ are the uptake rates of glucose, xylose, and oxygen, respectively. *K*_g_, *K*_z_, and *K*_o_ are the half-saturation constants and *K*_ie_ is the ethanol inhibition constant.

The dynamic mass balances for the extracellular environment are described by the usual ordinary differential equations:

(3) $$\begin{array}{c} \frac{dX_{\text{SC}}}{dt}=\mu_{\text{SC}}X_{\text{SC}},\\ \frac{dX_{\text{EC}}}{dt}=\mu_{\text{EC}}X_{\text{EC}},\\ \frac{dG}{dt}=-v_{\text{g},\text{SC}}X_{\text{SC}},\\ \frac{dZ}{dt}=-v_{\text{z},\text{EC}}X_{\text{EC}},\\ \frac{dE}{dt}=v_{\text{e},\text{SC}}X_{\text{SC}}+v_{\text{e},\text{EC}}X_{\text{EC}}, \end{array}$$

where *X* is the cellmass concentration and *v*_e_ is the ethanol exchange flux from microbial species. Extracellular oxygen balances are omitted on the assumption that direct manipulation of dissolved oxygen is possible. The dissolved oxygen (DO) concentration is represented as the percent of saturation (*O*/*O*_sat⁡_), where *O*_sat⁡_ is the oxygen saturation concentration. It is reported by Lisha and Sarkar [13] that if the oxygen concentration is higher than 25% of the saturated concentration, the ethanol production is practically insensitive to oxygen concentration in the medium. For all aerobic simulations, the dissolved oxygen is considered to be regulated at 0.29 mM, which corresponds to 98% of the saturated oxygen concentration.

### 2.2. Model Parameters and Dynamic Simulation

All the simulations are performed in Matlab environment using ode23 to integrate the extracellular dynamic mass balance equations and the COBRA Toolbox [19] with Matlab interface to the GNU linear program code* glpk* to solve the inner linear program. The substrate uptake parameters and the operating conditions used for all the dynamic simulations are listed in Table 1. The differences between the substrate uptake rates under aerobic and anaerobic conditions are neglected. The final batch time is chosen as the time when the glucose concentration dropped below 0.1 g/L. Since the optimal growth rate is being determined by solving a linear program, there may exist many different flux distributions that produce the same optimal growth rate. The problem of such multiple optimal solutions with respect to ethanol production rate is checked by first solving the linear program for maximization of cellmass and then by constraining the cellmass at this maximum value and solving the linear program again for maximum ethanol production rate.

Batch ethanol productivity (Pr_eth_) is defined as the overall rate of ethanol production:

(4) $$\begin{array}{c} \text{P}{\text{r}}_{\text{eth}}=\frac{{\left(EV\right)}_{t_{\text{f}}}}{t_{\text{f}}}, \end{array}$$

where *t*_f_ is the fermentation time.

The switching time (*t*_s_) for aerobic to anaerobic condition for fixed final time *t*_f_ is determined optimally by solving the following single variable optimization problem using the bounded search algorithm fminbnd in Matlab:

(5) Maximizets EVtftfSubject  to:    dFBA  model     l tLB≤ts≤tUB,

where *t*_LB_ and *t*_UB_ are appropriate lower and upper bounds for switching time, respectively.

### 2.3. Genetic Engineering Strategies for Enhanced Ethanol Production

*E. coli* can naturally convert both hexose and pentose sugars into ethanol via a heterofermentative process [20]. However, the native ethanol production pathway is suboptimal because of other fermentation products such as acetate, formate, lactate, and succinate. Redirecting the flow of carbon going to cellmass or these by-products towards ethanol will result in increased ethanol yield. Trinh et al. [21] reported the genetic engineering for minimal* E. coli* cell which can produce ethanol most efficiently from hexose and pentose sugars. Recently Kim and Reed [22] reported a number of genetic engineering strategies for enhanced ethanol production by* E. coli* from glucose and the strategies involve deletion of double, triple, quadruple, and quintuple genes. We choose a set of such gene deletion strategies on xylose-selective* E. coli* strain ZSC113 and investigate their potential for enhanced ethanol production from coculture fermentation of glucose/xylose mixtures. The details of these strategies are listed in Table 2.

Under anaerobic condition* S. cerevisiae* produces four major products from glucose: cellmass, ethanol, carbon dioxide, and glycerol. Redirecting the flow of carbon going to cellmass or glycerol towards ethanol will result in increased ethanol yield. There are efficient metabolic engineering strategies for redirecting carbon flux from glycerol to ethanol and they involve engineering of reactions with the cofactors NADH and/or NADPH in the cell. A number of genetic manipulation strategies for the engineering of redox metabolism in* S. cerevisiae* have been suggested by Bro et al. [12] and the efficiency of such manipulations for enhancing ethanol production has been analysed in the context of batch coculture fermentation of substrate selective microbes by Lisha and Sarkar [13]. We select two good strategies on* S. cerevisiae* and perform coculture fermentation with engineered ZSC113. The genetic engineering strategies on* S. cerevisiae* are insertion of NADP dependent glycerol 3-phosphate dehydrogenase (R00845) and insertion of nonphosphorylating NADP dependent glyceraldehyde-3-phosphate dehydrogenase (R01058). The details of the metabolic engineering strategies are listed in Table 3.

## 3. Results and Discussion

**Figure 1:**
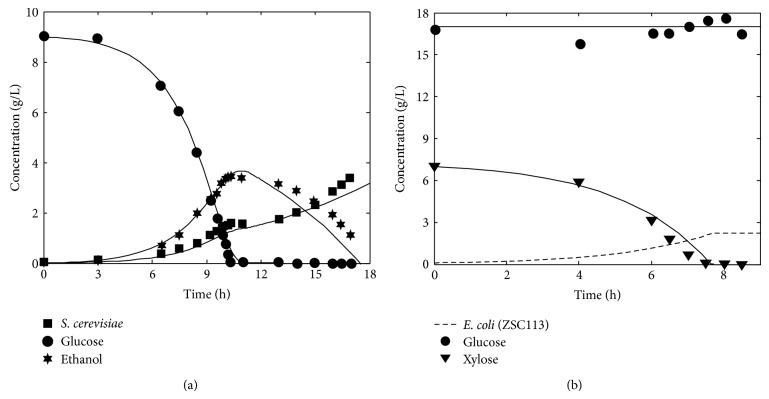
Comparison of model predictions and experimental data for aerobic batch culture of (a) wild-type* S. cerevisiae* [23] and (b) recombinant* E. coli* strain ZSC113 [24]. Experimental data are indicated by symbols and model predictions by lines.

In order to assess the validity of the developed dFBA model, we compare the model predictions for measurable state variables against experimental observations collected from the literature and Figure 1 shows the validation results.  Figure 1(a) shows the comparison of dFBA model predictions of wild-type* S. cerevisiae* under aerobic batch culture with experimental data taken from Jones and Kompala [23]. Operating conditions such as initial glucose concentration, initial cellmass concentration, and fermentation time are taken from the experimental information. It may be noted that the model predictions and experimental data show reasonably good agreement for cellmass production and glucose consumption. Similarly, the model also predicts the sequential production and consumption of ethanol by* S. cerevisiae* under aerobic condition.  Figure 1(b) shows the comparison of dFBA model predictions for aerobic batch culture of recombinant* E. coli* strain ZSC113 against experimental data taken from Eiteman et al. [24]. The model is capable of predicting the xylose selectivity of ZSC113 as shown in experimental data. Dynamic FBA model predictions of batch coculture of wild-type* S. cerevisiae* and xylose-selective* E. coli* strain ZSC113 are experimentally validated by Hanly et al. [11]. It may be noted that the predictions of the model are in good agreement with experimental observations under various conditions. Therefore, the validated dFBA model can provide the basis for performing various* in silico* metabolic engineering experiments and deriving strategies for enhanced ethanol production.

### 3.1. Coculture of Wild-Type* S. cerevisiae* and Genetically Modified* E. coli* Strain ZSC113

**Figure 2:**
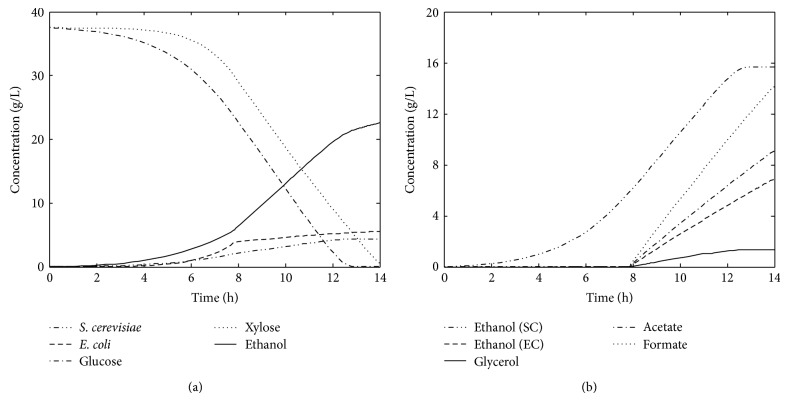
Batch coculture simulation of wild-type* S. cerevisiae* and recombinant* E. coli* strain ZSC113 on 50/50 glucose/xylose (%/%) mixture (SC:* S. cerevisiae*; EC:* E. coli* strain ZSC113).

The batch coculture simulations are carried out with wild-type* S. cerevisiae* that selectively consumes only glucose and engineered* E. coli* strain ZSC113 that consumes only xylose. The kinetic parameters and operating conditions used for the simulations are given in Table 1. The ratio of inoculum concentration and the fermentation time are determined by a systematic sensitivity analysis such that both the sugars are almost (99%) consumed by the end of fermentation [13].  Figure 2 shows the result of batch coculture fermentation with 50/50 glucose/xylose (%/%) mixture. This batch coculture (hereafter referred to as base case) for a fermentation time of 14 h and an optimum aerobic to anaerobic switch (*t*_s_) at 7.8 h (determined by solving (5)) produces 22.6 g ethanol with an ethanol yield (*Y*_eth_) of 0.3 g/g and productivity (Pr_eth_) of 1.61 g/h. The contribution of ethanol from* S. cerevisiae* and ZSC113 is 15.7 and 6.9 g, respectively. Glycerol is produced as a by-product (1.34 g) from* S. cerevisiae* [25]. Similarly, acetate (9.08 g), formate (14.2 g), and succinate (0.06 g) are also produced as by-products from ZSC113 [24]. No lactate is produced as ethanol production is preferred over lactate production at the optimal growth condition [22].

**Figure 3:**
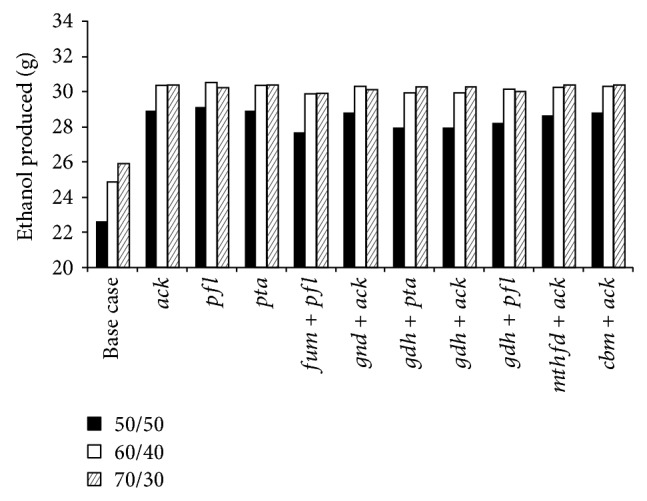
Model predictions of the amount of ethanol produced from various glucose/xylose mixtures during batch coculture fermentations of wild-type* S. cerevisiae* and ZSC113 with additional genetic manipulations (base case: wild-type* S. cerevisiae* and ZSC113).

The effect of substrate composition on batch ethanol productivity is investigated by simulations with 60/40 and 70/30 glucose/xylose mixtures by keeping the total amount of sugar the same as that in 50/50 mixture. However, the increase in glucose concentration in the substrate requires a corresponding increase in fermentation time to 16 h for 60/40 mixture and 17 h for 70/30 mixture. The results are shown in Figure 3. The 60/40 mixture for a batch time of 16 h and *t*_s_ of 6.9 h produces 24.8 g ethanol with *Y*_eth_ of 0.33 g/g and Pr_eth_ of 1.55 g/h, respectively. Amount of ethanol produced by* S. cerevisiae* is 18.8 g and that by ZSC113 is 5.97 g. For a batch time of 17 h (*t*_s_ = 6.2 h), 70/30 mixture produces 25.9 g ethanol with *Y*_eth_ and Pr_eth_ of 0.35 g/g and 1.53 g/h, respectively. The contribution of ethanol from* S. cerevisiae* is 21.3 g and from ZSC113 is 4.59 g. The amount of ethanol thus increases with increase in glucose concentration in the mixture as reported in the case of genetically engineered xylose-fermenting* S. cerevisiae* [26]. However, the Pr_eth_ decreases marginally due to the increase in fermentation time. Amount of glycerol produced from* S. cerevisiae* as a by-product increases with increase in glucose concentration in the substrate (1.92 g and 2.36 g, respectively, from 60/40 and 70/30 mixtures). On the other hand, production of acetate, formate, and succinate from ZSC113 decreases with increase in glucose concentration (decrease in xylose concentration) in the substrate. The 60/40 mixture produces 7.86 g acetate, 12.3 g formate, and 0.05 g succinate while 70/30 mixture produces 5.99 g acetate, 9.29 g formate, and 0.04 g succinate.

**Figure 4:**
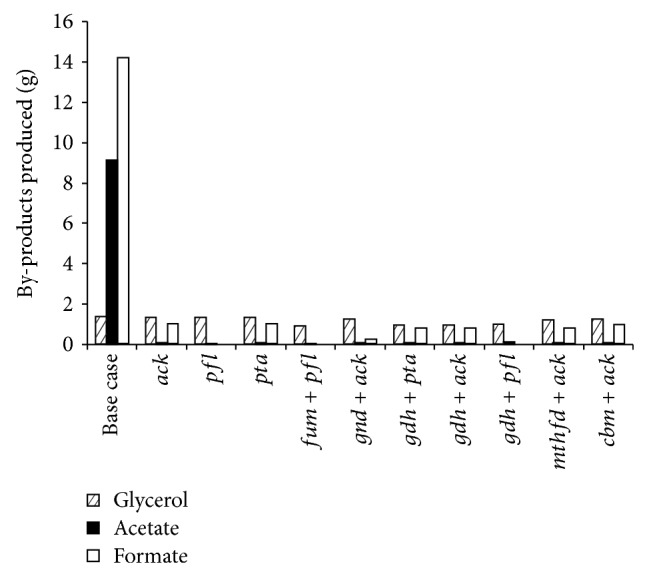
Model predictions of the amount of by-products produced from* S. cerevisiae* and xylose-selective* E. coli* strain ZSC113 during batch coculture fermentation of 50/50 glucose/xylose (%/%) mixture using wild-type* S. cerevisiae* and ZSC113 with additional genetic manipulations (base case: wild-type* S. cerevisiae* and ZSC113).

The effect of genetic manipulations on ZSC113 during coculture fermentation is shown in Figure 3 for various glucose/xylose mixtures. It may be noted that enhanced production of ethanol is predicted for all the manipulations. Among the gene deletion strategies studied here, the single gene deletion strategies seem to be more effective than double gene deletion strategies. The deletion of acetate kinase gene (Δ*ack*) enhances the production of ethanol to 28.9 g (*Y*_eth_ of 0.39 g/g and Pr_eth_ of 2.06 g/h) for 50/50 glucose/xylose (%/%) mixture, where the amounts of ethanol produced by* S. cerevisiae* and ZSC113 are 15.7 g and 13.2 g, respectively. Thus strategy Δ*ack* increases the contribution of ZSC113 twofold. The enhancement in *Y*_eth_ and Pr_eth_ is 30% and 28.2%, respectively, with respect to the base case.  Figure 4 shows that strategy Δ*ack* reduces the formation of acetate and formate as by-products significantly (0.05 g and 0.97 g, resp.). The reduction in by-products is responsible for enhanced production of ethanol as the carbon flux is now redirected from these by-products to ethanol. Deletion of the gene pyruvate formate lyase (Δ*pfl*) enhances the production of ethanol to 29.1 g, where the amounts of ethanol produced by* S. cerevisiae* and ZSC113 are 15.7 g and 13.4 g, respectively. Thus strategy Δ*pfl* also increases the contribution of ZSC113 twofold. The *Y*_eth_ and Pr_eth_ are 0.4 g/g and 2.08 g/h, respectively, which correspond to 31.7% enhancement in *Y*_eth_ and 29.2% enhancement in Pr_eth_ compared to the base case. Strategy Δ*pfl* reduces acetate formation to 0.03 g and formate production as a by-product is completely eliminated. Deletion of phosphotransacetylase (Δ*pta*) has the same effect as Δ*ack*. The other manipulations involving double gene deletions also result in enhanced production of ethanol (Figure 3) and corresponding reduction in by-products formation. The strategies involving* pfl* (Δ*fum* + Δ*pfl* and Δ*gdh* + Δ*pfl*) eliminate the production of formate completely (Figure 4). The various double gene deletion strategies produce ethanol between 27.6 g and 28.8 g for 50/50 mixture. Studies on 60/40 and 70/30 mixtures reveal that all the gene deletion strategies are equally effective for such mixtures as they enhance the ethanol production to almost the same extent.

There are some experimental results available in the literature on enhanced ethanol production by genetically engineered* E. coli*. Ma et al. [27] performed metabolic engineering study on an ethanol-tolerant* E. coli* MG1655 strain for enhanced ethanol production from glucose and xylose. Knockout of pyruvate formate lyase (*pflB*) and lactate dehydrogenase (*ldhA*) genes and expression of* Zymomonas mobilis* alcohol dehydrogenase II (*adhB*) and pyruvate decarboxylase (*pdc*) genes in the ethanol-tolerant mutant resulted in the production of ethanol as a principal fermentation product. Such a strain having double knockout and gene expressions produced 41.6 g/L ethanol from 100 g/L glucose and 35.8 g/L ethanol from 100 g/L xylose. This corresponds to 37 and 36.5% enhancement in ethanol production, respectively, with respect to the parent strain. The mutant also produced very little acetic acid and no formic acid and lactic acid. As noted in previous paragraph, our dFBA analysis of coculture system with ZSC113 strain having knockout at* pfl* gene also predicts 31.7% enhancement in *Y*_eth_ with respect to the base case, a reduction in acetic acid production, and complete elimination of formic acid and lactic acid as by-products. It is reported that recombinant* E. coli* strain FBR3 having knockout at* pfl* and* ldh* genes and expression of PET operon resulted in selective ethanol production from glucose, xylose, arabinose, or a mixture of these sugars [28]. Such strains achieved *Y*_eth_ of around 90% of the theoretical maximum. The bioethanol production potential of recombinant* E. coli* strain FBR3 from corn fiber hydrolysates was investigated [29] and the optimized fermentation of such strains resulted in *Y*_eth_ of 0.46 g/g, which is 90% of maximum theoretical value. *Y*_eth_ of 0.5 g/g was reported by Saha and Cotta [30] for recombinant* E. coli* strain FBR5 during the fermentation of wheat straw hydrolysates and the *Y*_eth_ is 98% of maximum theoretical value. High level expression of* adhII* and* pdc* genes from* Z. mobilis* in* E. coli* and the knockout of fumarate reductase (*frd*) gene have resulted in increased growth rate and ethanol production [31]. The developed strain completely eliminated the production of succinate and achieved *Y*_eth_ which is 103–106% of maximum theoretical value from glucose and xylose.

**Figure 5:**
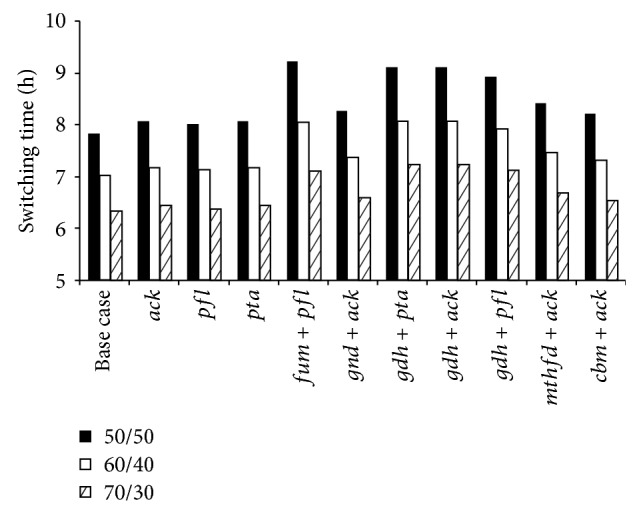
Model predictions of the optimum switching times for batch coculture fermentations of wild-type* S. cerevisiae* and ZSC113 with additional genetic manipulations (base case: wild-type* S. cerevisiae* and ZSC113).

Figure 5 shows the variation in optimal aerobic to anaerobic switching times (*t*_s_) for batch coculture fermentation using various glucose/xylose (%/%) mixtures. For 50/50 mixture coculture fermentation of wild-type* S. cerevisiae* and xylose-selective* E. coli* strain ZSC113 requires a *t*_s_ of 7.8 h. Fermentations with gene deleted microbes require higher switching times. Double gene deleted microbes require relatively higher switching times than single gene deleted microbes. Optimum switching times for all single gene deletion cases (Δ*ack*, Δ*pfl,* and Δ*pta*) are around 8 h for 50/50 mixture. Among the double gene deletion cases, the manipulations Δ*gnd* + Δ*ack*, Δ*mthfd* + Δ*ack,* and Δ*cbm* + Δ*ack* require almost the same *t*_s_ (around 8.3 h for 50/50 mixture). However, the manipulations Δ*fum* + Δ*pfl*, Δ*gdh* + Δ*pta*, Δ*gdh* + Δ*ack,* and Δ*gdh* + Δ*pfl* require significantly higher *t*_s_ (around 9 h for 50/50 mixture). This may be explained by reduced xylose uptake by these microbes. Up to 8 h of fermentation, these engineered microbes consume only 15% of total xylose and produce only 2.8 g/L of ZSC113 cellmass. But microbes engineered by all other strategies consume around 23% xylose and produce around 4.4 g/L of ZSC113 during the aerobic phase. The consumption of glucose (~40%) and the formation of* S. cerevisiae* (~2.2 g/L) are comparable for all the gene deletion strategies. Under the condition of reduced xylose consumption compared to other strategies, the aerobic phase needs to be sufficiently extended for the formation of enough ZSC113 cellmass and this may be the reason for the significantly higher *t*_s_ for these strategies. Investigations on 60/40 and 70/30 mixtures predict the same trend, but the switching times decrease with increase in glucose concentration in the substrate. Lisha and Sarkar [13] also observed the reduction in *t*_s_ with increase in glucose concentration in the substrate for* in silico* coculture of* E. coli* strain ZSC113 with genetically modified* S. cerevisiae*.

**Figure 6:**
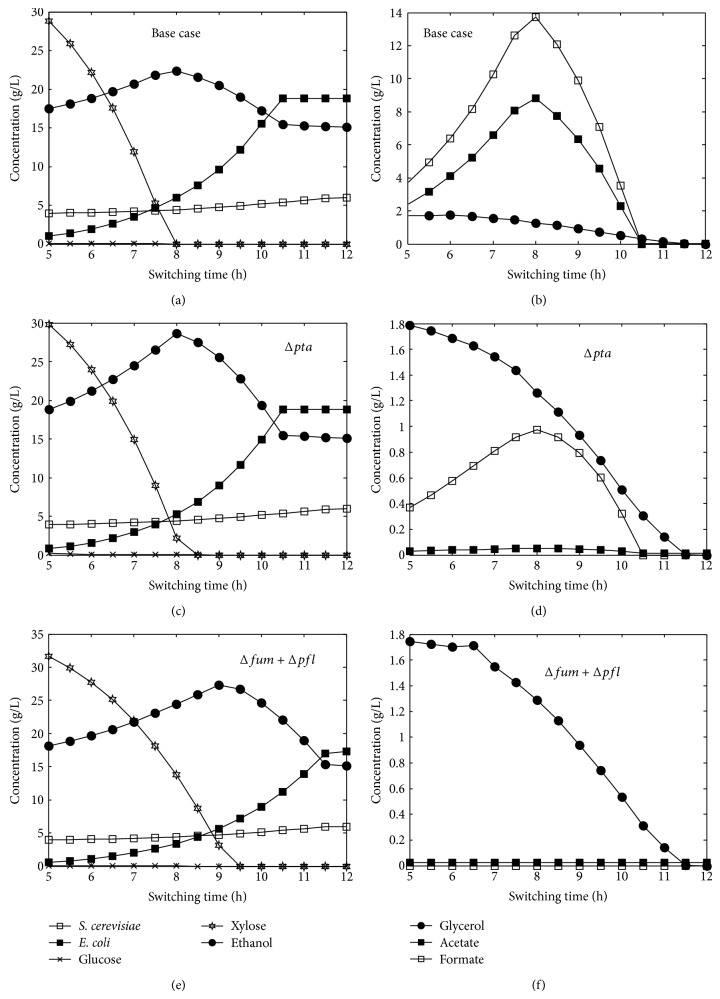
Model predictions of the effect of switching times on batch coculture fermentation of 50/50 glucose/xylose (%/%) mixture using wild-type* S. cerevisiae* and ZSC113 with additional genetic manipulations (base case: wild-type* S. cerevisiae* and ZSC113). The concentrations plotted are the concentrations at final time (14 h).

The effect of aerobic to anaerobic switch on batch coculture performance of base case and systems with genetically modified ZSC113 strains is shown in Figure 6 for 50/50 glucose/xylose mixture. For all the cases, ethanol production increases with increase in switching time and reaches a maximum value and then decreases. It is clear from the figure that a lower aerobic to anaerobic switch is associated with limited consumption of xylose and complete consumption of xylose takes place near optimal switching time. Glucose consumption by* S. cerevisiae* is practically insensitive to switching time in the range studied and it might be due to the potential of* S. cerevisiae* to grow and produce ethanol under both aerobic/anaerobic conditions. The longer aerobic phase is associated with reduced production of by-products from both* S. cerevisiae* and ZSC113. Although growth of* S. cerevisiae* is not very sensitive to switching time, the ZSC113 grows much more rapidly in the aerobic phase. So the optimal switching time strikes the correct balance between growth of microorganisms and product formation.

### 3.2. Coculture of Genetically Modified* S. cerevisiae* and* E. coli* Strain ZSC113

**Figure 7:**
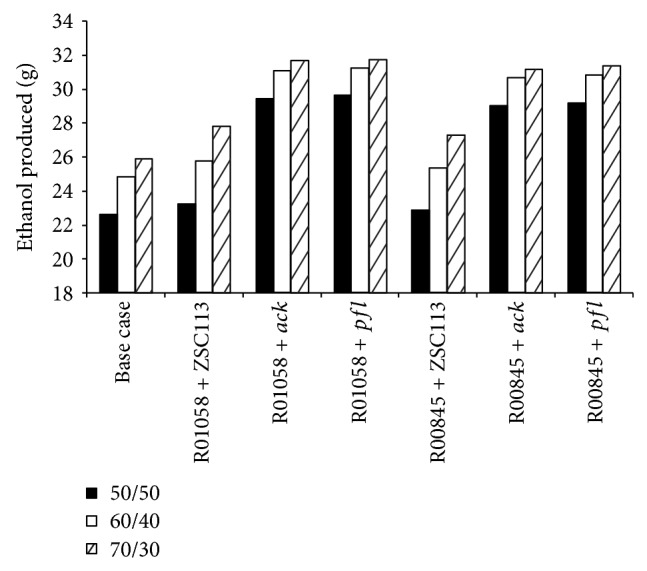
Model predictions of the amount of ethanol produced from various glucose/xylose mixtures during batch coculture fermentations of wild-type* S. cerevisiae* and ZSC113 with genetic manipulations on both microorganisms (base case: wild-type* S. cerevisiae* and ZSC113).

**Figure 8:**
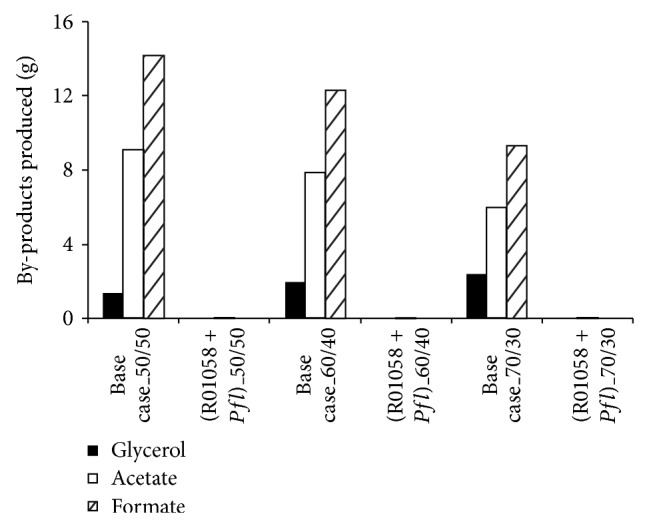
Model predictions of the amount of by-products produced from various glucose/xylose mixtures during batch coculture fermentations of wild-type* S. cerevisiae* and ZSC113 with genetic manipulations on both microorganisms (base case: wild-type* S. cerevisiae* and ZSC113).

Lisha and Sarkar [13] recently reported the effect of various genetic engineering strategies on* S. cerevisiae* and classified the 8 gene insertion strategies into two groups (Group A1 and Group A2) based on the ethanol production in batch coculture fermentation with xylose-selective* E. coli* strain ZSC113. For a 60/40 glucose/xylose (%/%) mixture the manipulations belonging to Group A1 and Group A2 resulted in 3.87% and 1.93% enhancement in Pr_eth_ with respect to the base case. We select one strategy from each group as a representative (R01058 from Group A1 and R00845 from Group A2) and perform coculture fermentation with two recombinant ZSC113 (Δ*ack* and Δ*pfl*) that perform well when cocultured with wild-type* S. cerevisiae*. Combinations of genetic modifications that we investigate are R01058 + Δ*ack*, R01058 + Δ*pfl*, R00845 + Δ*ack,* and R00845 + Δ*pfl* for various glucose/xylose mixtures. The results are shown in Figure 7 and Table 4. It may be noted that strategies R01058 + Δ*ack* and R01058 + Δ*pfl* enhance the ethanol production when compared to strategy Δ*ack* or Δ*pfl* alone as discussed in Section 3.1. For a 50/50 mixture the coculture of* S. cerevisiae* plus R01058 and ZSC113 plus Δ*pfl* produces 29.6 g ethanol (*t*_s_ of 8 h) with *Y*_eth_ and Pr_eth_ of 0.4 g/g and 2.12 g/h, respectively. Amount of ethanol produced by* S. cerevisiae* is 16.3 g and that by ZSC113 is 13.3 g. The resultant enhancement in *Y*_eth_ and Pr_eth_ is 33.3% and 31.1%, respectively, with respect to the base case. Formation of glycerol and formate as by-products is completely eliminated by this genetic engineering strategy (Figure 8). Similarly, formation of acetate (0.03 g) as a by-product from ZSC113 also reduces significantly. The enhancement in *Y*_eth_ due to the genetic modification of* S. cerevisiae* by the insertion of R01058 is only 2.03% compared to the base case, while the enhancement in *Y*_eth_ is 31.7% due to genetic modification of ZSC113 by deletion of* pfl* gene. The genetic modification of ZSC113 has much more dominant effect on enhancement in ethanol production compared to the genetic modification of* S. cerevisiae*. It is mainly due to the redirection of carbon flux from significant amount of by-products acetate (9.08 g) and formate (14.2 g) to ethanol in ZSC113, while, in* S. cerevisiae*, the formation of glycerol as a by-product is limited (1.34 g). Other genetic modifications such as R01058 + Δ*ack*, R00845 + Δ*ack,* and R00845 + Δ*pfl* also cause similar enhancement in ethanol production for 50/50 mixture with *t*_s_ around 8 h for all cases. Studies on 60/40 and 70/30 mixtures show the same trend in amount of ethanol and by-products produced, as evident from Table 4 and Figure 8. Production of bioethanol steadily increases with increase in glucose concentration in the substrate. The genetic modification R01058 + Δ*pfl* yields 31.2 g ethanol (*Y*_eth_ of 0.42 g/g and Pr_eth_ of 1.95 g/h) for 60/40 mixture and 31.8 g ethanol (*Y*_eth_ of 0.42 g/g and Pr_eth_ of 1.87 g/h) for 70/30 mixture.

There are some experimental results available in the literature on gene insertion in* S. cerevisiae* for enhanced bioethanol production. Bro et al. [12] reported the* in vivo* testing of bioethanol production by expressing a gene encoding GAPN (nonphosphorylating NADP dependent glyceraldehyde-3-phosphate dehydrogenase, R01058) in wild-type* S. cerevisiae*. Anaerobic batch cultivation of the strain on glucose media resulted in *Y*_eth_ of 0.42 g/g and glycerol yield of 0.046 g/g. This corresponds to 2.44% enhancement in *Y*_eth_ and 43.2% reduction in glycerol yield, respectively, with respect to the parent strain. The recombinant* S. cerevisiae* (consumes both glucose and xylose) with expression of GAPN resulted in *Y*_eth_ of 0.36 g/g and glycerol yield of 0.018 g/g. This genetic modification enhances *Y*_eth_ by 24.1% and reduces glycerol yield by 59.1%. It is also reported that heteroexpression of GAPN and knockout of* FPS1* gene (a glycerol facilitator) in* S. cerevisiae* produces 9.18% more ethanol and 21.5% less glycerol compared to the parent strain from glucose media [32]. As noted in previous paragraph, our dFBA analysis also predicts enhancement in *Y*_eth_ for coculture fermentation with GAPN (R01058) inserted* S. cerevisiae* (instead of wild-type* S. cerevisiae*) and ZSC113 with* pfl* or* ack* gene deletion. The coculture of GAPN (R01058) inserted* S. cerevisiae* and ZSC113 with* pfl* gene deletion predicts 2.03% enhancement in *Y*_eth_ and the coculture of GAPN (R01058) inserted* S. cerevisiae* and ZSC113 with* ack* gene deletion predicts 1.8% enhancement in *Y*_eth_.

## 4. Conclusions

A detailed* in silico* analysis of the effect of a set of metabolic engineering strategies on* E. coli* strain ZSC113 has been studied in the context of batch coculture fermentation. The coculture process involves wild-type* S. cerevisiae* that natively consumes only glucose and recombinant* E. coli* strain ZSC113 that consumes only xylose. For 50/50 glucose/xylose (%/%) mixture, the deletion of gene* pfl* results in 31.7% enhancement in *Y*_eth_ and 29.2% enhancement in Pr_eth_ with respect to the base case (coculture fermentation of wild-type* S. cerevisiae* and ZSC113 without genetic modification). This is possible due to the redirection of carbon flux from cellmass, by-products like acetate and formate, to ethanol. Other manipulations also result in a similar enhancement in *Y*_eth_ and Pr_eth_ for various glucose/xylose mixtures. Genetic modifications on both microorganisms further enhance the bioethanol production potential of coculture fermentation. Coculture of* S. cerevisiae* plus R01058 and ZSC113 plus Δ*pfl* enhances *Y*_eth_ by 33.3% and Pr_eth_ by 31.1% compared to the base case for a 50/50 glucose/xylose mixture. Significant reduction in the formation of by-products (glycerol from* S. cerevisiae* and acetate/formate from* E. coli*) is responsible for this. For enhanced ethanol production from coculture system of substrate-selective microbes (glucose-selective* S. cerevisiae* and xylose-selective* E. coli* strain ZSC113) the genetic modification on ZSC113 is advantageous over genetic modification on* S. cerevisiae*.

A validated genome-scale model is an effective tool for metabolic phenotype studies. The simulations carried out in the present study predict potential genetic engineering strategies for enhancement of strain performance during coculture fermentation. Genome-scale metabolic engineering experiments are time consuming and the identification of appropriate genetic manipulations to be applied to an organism is an important challenge. Therefore, the results of* in silico* analysis provide valuable guidance for conducting* in vivo* experiments and also reduce the number of such expensive and time-consuming experimental trials.

## Figures and Tables

**Table 1:** Kinetic parameters and operating conditions for batch coculture simulations [13].

Parameter	Coculture		Parameter	Glucose/xylose (%/%)		
	*S. cerevisiae *	*E. coli *		50/50	60/40	70/30
*v*_g,max_ (mmol/g/h)	22.4	0	*G*_0_ (g/L)	37.5	45	52.5
*K*_g_ (g/L)	0.8	0	*Z*_0_ (g/L)	37.5	30	22.5
*v*_o,max_ (mmol/g/h)	2.5	20	*t*_f_ (h)	14	16	17
*K*_o_ (mM)	0.003	0.024				
*V*_z,max_ (mmol/g/h)	0	12				
*K*_z_ (g/L)	0	0.25				
*K*_i,e_ (g/L)	10	20				
*X*_0_ (g/L)	0.044	0.006				

**Table 2:** Metabolic engineering strategies for ethanol overproduction by recombinant *E.  coli* strain ZSC113 [22].

Number	Metabolic engineering strategy	Deleted reaction
1	Deletion of acetate kinase (Δ*ack*)	Acetate + ATP *↔* Acetyl phosphate + ADP

2	Deletion of pyruvate formate lyase (Δ*pfl*)	Acetyl-CoA + Formate → CoA + Pyruvate

3	Deletion of phosphotransacetylase (Δ*pta*)	Acetyl-CoA + Phosphate *↔* Acetyl phosphate + CoA

4	Deletion of fumarase (Δ*fum*) + Δ*pfl *	Fumarate + H_2_O *↔* L-malate Acetyl-CoA + Formate → CoA + Pyruvate

5	Deletion of phosphogluconate dehydrogenase (Δ*gnd*) + Δ*ack *	6-Phospho-D-gluconate + NADP → Ribulose 5-phosphate + NADPH + CO_2_ Acetate + ATP *↔* Acetyl phosphate + ADP

6	Deletion of glutamate dehydrogenase (Δ*gdh*) + Δ*pta *	Glutamate + NADP + H_2_O *↔* 2-Oxoglutarate + NADPH + NH_3_ Acetyl-CoA + Phosphate *↔* Acetyl phosphate + CoA

7	Δ*gdh* + Δ*ack *	Glutamate + NADP + H_2_O *↔* 2-Oxoglutarate + NADPH + NH_3_ Acetate + ATP *↔* Acetyl phosphate + ADP

8	Δ*gdh* + Δ*pfl *	Glutamate + NADP + H_2_O *↔* 2-Oxoglutarate + NADPH + NH_3_ Acetyl-CoA + Formate → CoA + Pyruvate

9	Deletion of methylenetetrahydrofolate dehydrogenase (Δ*mthfd*) + *∆ack *	5,10-Methylenetetrahydrofolate + NADP *↔* 5,10-Methenyltetrahydrofolate + NADPH Acetate + ATP *↔* Acetyl phosphate + ADP

10	Deletion of carbamate kinase (Δ*cbm*) + Δ*ack *	ATP + CO_2_ + NH_4_ *↔* ADP + Carbamoyl phosphate Acetate + ATP *↔* Acetyl phosphate + ADP

**Table 3:** Metabolic engineering strategies for ethanol overproduction by *S.  cerevisiae* [12].

Number	Metabolic engineering strategy	Inserted reaction
1	Insertion of NADP dependent glycerol 3-phosphate dehydrogenase (R00845)	Glycerol 3-phosphate + NADP *↔* D-Glyceraldehyde 3-phosphate + NADPH

2	Insertion of nonphosphorylating NADP dependent glyceraldehyde-3-phosphate dehydrogenase (R01058)	D-glyceraldehyde 3-phosphate + NADP → 3-phospho-D-glycerate + NADPH

**Table 4:** Ethanol yield and productivity of batch coculture fermentations with genetic modification on both microorganisms.

Strategy	Ethanol yield (*Y*_eth_), g/g			Ethanol productivity (Pr_eth_), g/h		
	Glucose/xylose (%/%)			Glucose/xylose (%/%)		
	50/50	60/40	70/30	50/50	60/40	70/30
R01058 + Δ*ack *	0.399	0.424	0.433	2.101	1.941	1.865
R01058 + Δ*pfl *	0.403	0.427	0.424	2.116	1.952	1.867
R00845 + Δ*ack *	0.395	0.418	0.421	2.072	1.916	1.833
R00845 + Δ*pfl *	0.398	0.420	0.429	2.082	1.924	1.846

R01058 + ZSC113	0.312	0.345	0.374	1.657	1.608	1.635
R00845 + ZSC113	0.306	0.339	0.367	1.631	1.582	1.605
*S. cerevisiae* (wild-type) + ZSC113	0.303	0.331	0.345	1.613	1.550	1.523
